# Cyclosporin A-mediated translocation of HuR improves MTX-induced cognitive impairment in a mouse model via NCOA4-mediated ferritinophagy

**DOI:** 10.18632/aging.205195

**Published:** 2023-11-09

**Authors:** Huang Ding, Rong Xiang, Yifan Jia, Jishi Ye, Zhongyuan Xia

**Affiliations:** 1 Department of Pain, Renmin Hospital of Wuhan University, Wuhan 430060, Hubei, People’s Republic of China; 2 Department of Otolaryngology-Head and Neck Surgery, Renmin Hospital of Wuhan University, Wuhan 430060, Hubei, People’s Republic of China

**Keywords:** CICI, HuR, cyclosporin A, NCOA4, ferritinophagy

## Abstract

Chemotherapy-induced cognitive impairment (CICI) is a subject that requires critical solutions in neuroscience and oncology. However, its potential mechanism of action remains ambiguous. The aim of this study was to investigate the vital role of HuR in the neuroprotection of cyclosporin A (CsA) during methotrexate (MTX)-induced cognitive impairment.

A series of Hu-antigen R (HuR) gain and loss experiments were used to examine cyclosporin A (CsA)-mediated translocation of HuR’s ability to improve MTX-induced cognitive impairment through NCOA4-mediated ferritinophagy *in vitro* and *in vivo*.

Obtained results show that the administration of CsA alleviated MTX-induced cognitive impairment in mice. The presence of MTX promoted the shuttling of HuR from the cytoplasm to the nucleus, whereas treatment with CsA increased cytoplasmic HuR expression levels and the levels of ferritinophagy-related proteins, such as NCOA4 and LC3II, compared to the MTX group. However, applying KH-3, an inhibitor of HuR, reversed CsA’s impact on the expression of ferritinophagy-related proteins in the hippocampus and *in vitro*. Also, treatment with CsA attenuated microglial activation by altering Iba-1 expression and decreased TNF-α and IL-1β levels in mice hippocampi. Moreover, KH-3 neutralized CsA’s effects on the expression of both Iba-1 and HuR *in vivo* and *in vitro*.

In summary, CsA was confirmed to have a neuroprotective role in CICI. Its possible underlying mechanisms may be involved in the translocation of HuR. Mediating the translocation of HuR during CICI could mitigate neruoinflammation and neuronal apoptosis via NCOA4-mediated ferritinophagy and, thus, alleviate cognitive impairment in mice with CICI.

## INTRODUCTION

So far, chemotherapy is still the most crucial treatment for all kinds of tumors. While its use has seen a significant increase in the number of tumor survivors, that application has also caused a variety of adverse effects. Among these adverse effects is chemotherapy-induced cognitive impairment (CICI), often referred to as “chemobrain”, which has attracted widespread attention from clinical professionals [1]. The neuropsychological symptoms of CICI are centered around continuous memory impairment, attention loss, speech and visual spatial perception dysfunction, reduced motor speed and executive function, which seriously affect the biological functions and quality of life of patients with cancers.

In recent years, the widespread application of chemotherapy has countered increasing tumor incidence rates and prolonged the survival time of tumor patients, but it has also resulted in a considerable rise in the incidence of CICI. Moreover, more than three-quarters of tumor patients have long-term CICI, with some cases lasting for more than 10 years, hence, attracting the attention of patients and the society [1, 2]. Although some studies have associated the occurrence of CICI with the damage or remodeling of the neuronal structure, the key molecular mechanism of CICI’s development and action, especially the mechanism of its early occurrence, has not been elucidated. Additionally, effective and feasible prevention and treatment measures are still undetermined [3–5]. Therefore, understanding and further clarifying the pathogenesis of CICI is particularly important, as is early intervention to improve the prognosis and quality of life of patients with CICI. There is a need for urgency in the resolution of this in the field of neuroscience and oncology.

Hu-antigen R (HuR), also known as embryonic lethal abnormal vision-like 1 (ELAVL1), is an RNA-binding protein that has been evaluated extensively [6]. In its normal state in a cell, HuR is located primarily in the nucleus and is inactive. However, when a cell encounters a certain pathological environment, HuR will bind with various mRNAs in the nucleus and escort them to the cytoplasm to maintain the stability of the bound mRNAs and promote their translation process. Reportedly, HuR is involved in myocardial autophagy flux injuries, and this could become a new therapeutic target for hypoxia-induced myocardial injuries. The ELAVL1/HuR pathway also participates in NCOA4-mediated ferritinophagy, contributing to ferroptosis in liver fibrosis [7]. However, the specific role of HuR in CICI has not been explored yet. Cyclosporine A (CsA), an immunosuppressing drug, allegedly has a neuroprotective effect on brain injury and cognitive function [8] and possesses the ability to promote the shuttling of HuR from the nucleus to the cytoplasm [9]. Given these facts, it is reasonable to explore the specific role of CsA’s regulation of HuR in CICI.

In this study, animal models and neural cells were treated with MTX to establish a chemotherapy-induced cognitive impairment model. Using CsA intervention, the role of HuR was examined in the progression of MTX-induced neurotoxicity, as was the protective role of CsA against CICI in mice. Per the findings here, CsA is a potential therapeutic agent to target CICI, acting demonstrably by ameliorating NCOA4-mediated ferritinophagy via HuR.

## MATERIALS AND METHODS

### Animals

Seven-week-old male C57BL/6J mice were obtained from the Hubei Provincial Center for Disease Control and Prevention and maintained under standard conditions (12-h/12-h light/dark cycle, 25° C, 60% ± 10% humidity, and ad libitum access to food and water). After a one-week acclimation period, the mice were assigned randomly to one of five groups (n=8 per group): (a) control; (b) MTX treatment; (c) MTX+CsA treatment; (d) MTX+CsA+KH-3 treatment; (e) MTX+CsA+DMSO treatment. Mice in the MTX treatment group received intraperitoneal injections of MTX (100 mg/kg, dissolved in PBS) every five days for a total of three times. Mice in the MTX+CsA treatment group got the same MTX regimen and then CsA (20 mg/kg). Mice in the MTX+CsA+KH-3 treatment group were given MTX and CsA regimen (the same amounts as in the MTX+CsA group) plus intraperitoneal injections of KH-3 (50 mg/kg per day) for five days. The MTX+CsA+DMSO treatment group mice received the same MTX and CsA regimen (as the two previous groups) plus intraperitoneal injections of DMSO (50 mg/kg per day) for five days.

### Open field test

The open field test served to evaluate the locomotor activity (total distance traveled) and anxiety levels (time spent in the central area) of rodents in a novel environment and was conducted using a method similar to that reported previously [10], with a minor modification. A non-enclosed box with dimensions of 50 cm x 50 cm x 40 cm was used. Mice were placed in the center of the open field and allowed to explore for 5 minutes in a quiet and dimly lit environment. Each animal's behavior was tracked using a behavior-recording software to measure the total distance traveled, time spent in the center, and freezing behavior. Prior to testing, each mouse was cleaned thoroughly of previous odors and excretions using 75% ethanol.

### Novel object recognition test

The novel object recognition test was performed as described previously [11]. Two similar objects and one visibly different object were placed in an open field with 40 cm x 60 cm x 50 cm dimensions. For two consecutive days, mice were put individually in the empty testing field for 10 minutes. On the third day, the mice were exposed to the testing field with two similar objects for 10 minutes and, one hour later, to one of these objects and a new object that differed in both color and shape for another 10 minutes while their exploration time and discrimination index (time spent with the new object / (time spent with the new object + time spent with the familiar object) x 100%) were recorded with a video tracking system.

### Fear conditioning test

The fear conditioning test was carried out in two stages similar to that reported previously [11]: the conditioned fear training and the contextual fear testing. All the mice in this study participated in the behavioral experiments. The activity trajectories of the mice were traced automatically using an image monitoring system (XR-XC404).

The conditioned fear training was performed on the 2nd day after surgery. Here, mice were placed in a sound-isolated training chamber individually and allowed to acclimate for 180 s, followed by a 3 s tone test (75 dB, 3,000 Hz) and a 2 s foot shock period (0.75 mA, with the last 2 s of the tone period co-terminating with the shock) before they were returned to their home cages for 30 s. The contextual fear testing was performed 24 hours after the end of the training. In this case, mice were returned to the same experimental chamber without any stimuli, and their immobility time (defined as no activity other than respiration) during a 5-min period was recorded. At the end of each individual session, the testing chamber was immediately wiped clean with 75% alcohol and air-dried before the next mouse was tested.

### Western blot analysis

Western blot was used to evaluate the expression of HuR, NCOA4, FTH1, LC3I, ILC3II, and GAPDH proteins in total protein, as well as the HuR protein in both plasma and nuclear proteins in the hippocampus. Five hippocampal tissues were harvested from each group of mice, quantified using the BCA method, and subjected to electrophoresis with SDS-PAGE. Next, the proteins were transferred to a PVDF membrane using a semi-dry transfer system, after which the membrane was washed in a washing solution for 5 minutes, blocked with 5% skim milk on a shaker at room temperature for 2 hours, and incubated with primary antibodies against HuR (1:10000), NCOA4 (1:1000), FTH1 (1:2000), LC3 (1:1000), and GAPDH (1:10000) overnight at 4° C. On the next day, the membrane was washed and incubated with a secondary antibody (1:5000) at room temperature for 1 hour. After the incubation, the membrane was washed 3 times, and protein bands were visualized using an imaging system. The gray values of the protein bands were analyzed using ImageJ software, and the ratio of the gray value of the target protein to that of the internal reference protein was used to represent the relative expression levels of the target protein.

### Immunofluorescence microscopy

The expression of HuR and Iba-1 in hippocampal tissues was assessed using immunofluorescence. After sacrificing the mice, hippocampal tissues of each group were harvested and immersed in 4% paraformaldehyde, fixed overnight at 4° C, routinely embedded, sliced continuously, blocked with 5% bovine serum albumin (BSA) for 20 minutes, washed with PBS, and incubated with mouse anti-HuR (Abcam, UK) and Iba-1 antibody (Abcam, UK) overnight at 4° C. On the following day, the tissues were washed with PBS and incubated with secondary antibodies Alexa-Fluor 488 (green, 1:500, A-11029; Invitrogen, USA) and 594 (red, 1:500, A-11032; Invitrogen, USA) in the dark at room temperature for 2 hours and then stained with DAPI. One last wash with PBS was performed, and the tissues were observed and photographed with an inverted fluorescence microscope (Olympus Corporation, Japan). Image J software was used for the analysis of fluorescence intensity.

### ELISA

The levels of tumor necrosis factor-α (TNF-α) and interleukin-1β (IL-1β) in hippocampi and supernatants of cultured cells were measured utilizing ELISA kits (R&D Systems, USA). Absorbance was determined at 450 nm using a microplate reader. This assay was performed three times.

### TUNEL staining

TUNEL assay was performed on 4 μm paraffin-embedded hippocampal tissue sections using a TUNEL cell apoptosis detection kit. The tissue sections were deparaffinized in xylene, rehydrated, immersed in 3% H2O2 for 5 min, treated with a proteinase K solution on ice for 5 min, and incubated with 50 μL of TUNEL reaction mixture at 37° C for 60 min. They were then stained with DAB and counterstained with hematoxylin before TUNEL-positive cells in the hippocampi were observed under a light microscope – the percentage of TUNEL-positive cells was calculated as an indicator of cell apoptosis. The average value of three random fields selected from each section was determined. The TUNEL-positive cell percentage was calculated as TUNEL-positive cell number divided by total cell number, multiplied by 100%.

### Transmission electron microscopy (TEM)

Transmission electron microscopy was used to examine the ultrastructure of hippocampi. Freshly isolated hippocampal tissues were rinsed, fixed, dehydrated, embedded and sliced into 60-90 nm ultrathin sections. The sections were then stained with lead and double staining, and 3-5 fields of view were selected from each section for scrutiny of the size, shape, and ultrastructure of hippocampal neurons, as well as of cell membranes, nuclei, and mitochondrial structures, under a transmission electron microscope. Images were acquired using a Tecnai G2 20 TWIN transmission electron microscope.

### In vitro

Mouse hippocampal HT22 cell lines were purchased from Wuhan Hycell Biotech and cultured in a DMEM (Hycell Biotech, China) containing 10% FBS (Hycell Biotech, China), 2 mM glutamine and 200 mM streptomycin/penicillin (Invitrogen). The culture was kept at 90% ~ 95% relative humidity, 5% CO2 and 37° C, with the medium renewed every 3 days. HT22 cells were cultured in T-75 culture bottles and digested by trypsin and re-suspended in the medium when the density reached 80%~90%.

1*10^4^ HT22 cells were inoculated in 96-well plates for a 24h adherent culture and were transfected first with small interfering RNA (siRNA) before transfection with HuR-siRNA or non-targeting siRNA (RiboBio, China) for 48 h using a Lipofectamine 3000 reagent (Invitrogen, USA). The sequences of the siRNAs used were as follows: siRNA-HuR 5'-CGGTTTGGGCGAATCATCAACTCCA-3'; siRNA-NC 5'-CGGGTGGGCAACTTAACCATTTCCA-3'. After the transfection, HT22 cells were stimulated with MTX for 24 h and then treated with 50 ng/mL CsA for 1 h. Generally, treatment was performed based on whether the cells were categorized as being in the control group, MTX group (at 100 μM), MTX + CsA group + NC-siRNA, or MTX +CsA group + HuR-siRNA. HT22 cell stimulations with 100 μM MTX for 24 h was conducted based on previous literature reports on how to build a neurotoxicity model.

### Cell apoptosis detection

The cells in each group were washed, collected, and, according to the instructions that came with the Annexin V-FITC Apoptosis Kit (BD Biosciences, USA), were suspended in 300 μL of binding buffer and incubated with 50 μL Annexin V-FITC and 5 μL PI for 10 min and 5 min, respectively, in the dark, at room temperature. The apoptotic index was analyzed with FACSCalibur (BD Biosciences, USA) within 1 h. Flow cytometry was used to determine the cell apoptosis rate.

### Immunofluorescence co-localization analysis

HT22 cells were fixed with 4% paraformaldehyde for 30 min at room temperature, blocked with 1% BSA, and then immunostained with the following primary antibodies: NCOA4 (1:500) and HuR (1:1000) and secondary antibodies (mouse and rabbit) (1:1000). Fluorescent images were taken with a fluorescence microscope (Olympus Corporation, Japan). This assay was carried out three times. Representative images are shown in the figures.

### Statistical analysis

Data statistical analysis was performed using the Graph Pad Prism 8.4.3 software. Normally distributed quantitative data are presented as the mean ± standard deviation. The one-way analysis of variance (ANOVA) was used for comparisons between groups, and Tukey's test was deployed for pairwise comparisons. A p-value less than 0.05 was considered statistically significant.

### Availability of data and materials

The datasets analyzed during the current study are available from the corresponding author on reasonable request.

## RESULTS

### CsA alleviates MTX-induced cognitive impairment in mice

To evaluate MTX and CsA’s abilities to affect the cognitive behavior of mice in this study, a series of behavioral tests were performed, including the open field test, novel object recognition test and fear conditioning test, 3 days after the drug treatments.

**Figure 1:**
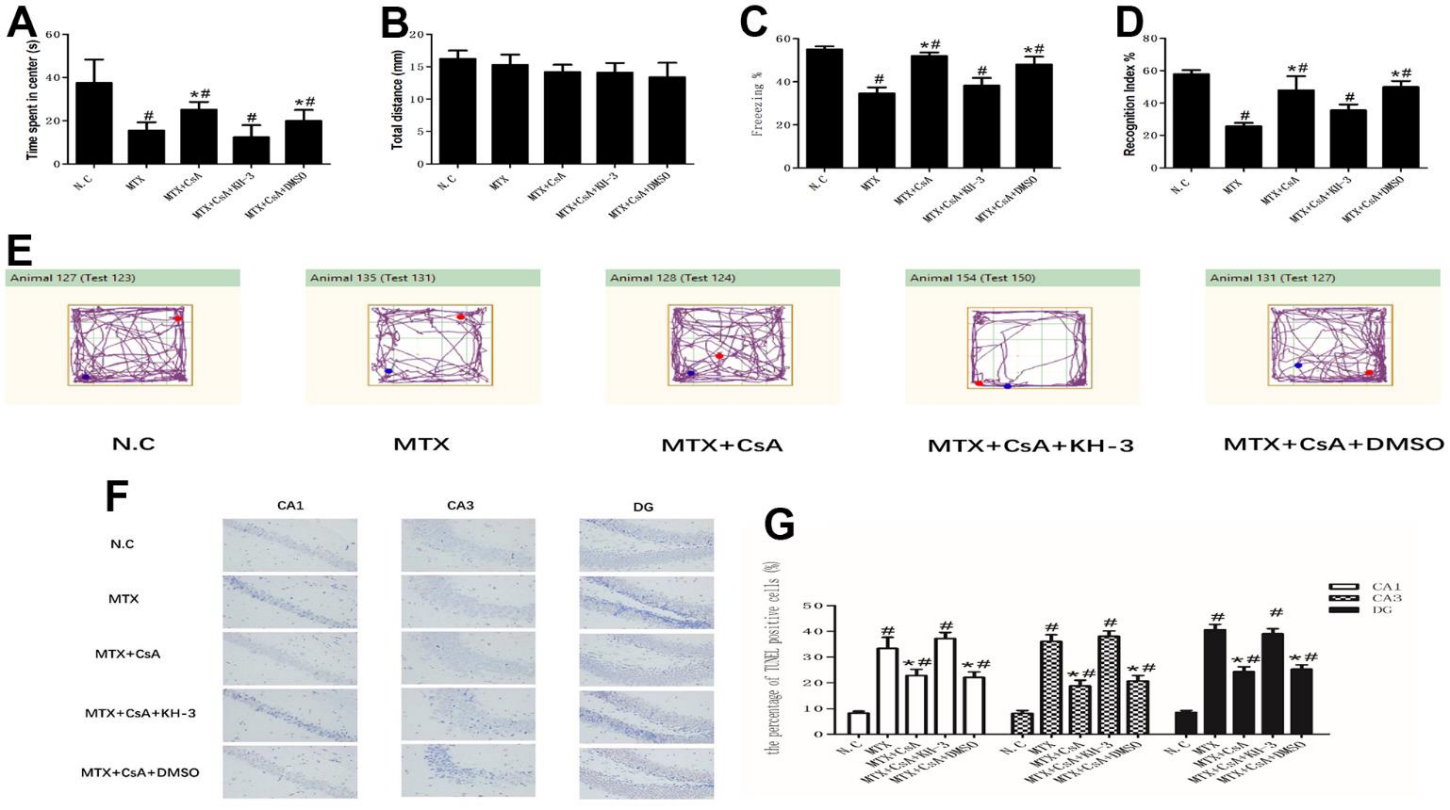
**Effects of CsA on MTX-induced cognitive impairment in mice.** (**A**) The total distance travelled during the initial 5 min of exposure to the training box in the open field test. (n=6 per group). #p <0.05 versus the control group; *p<0.05 versus the MTX group. (**B**) The time spent in the center of the open field during 5 min of exposure to the training box in the open field test. (n=6 per group). #p <0.05 versus the control group; *p<0.05 versus the MTX group. (**C**) The total percentage of freezing during the 5 min of contextual fear conditioning test (test phase of the FCT). (n=6 per group). #p <0.05 versus the control group; *p<0.05 versus the MTX group. (**D**) The recognition index of the novel object in the NOR. (n=6 per group). #p <0.05 versus the control group; *p<0.05 versus the MTX group. (**E**) Movement tracks showing 5 min of open field exploration by mice. (n=6 per group). #p <0.05 versus the control group; *p<0.05 versus the MTX group. (**F**) Neuronal apoptosis in the CA1, CA3 and DG regions of the hippocampus, as determined using Tunnel staining. Representative micrographs: ×400 magnification. (n=6 per group). #p <0.05 versus the control group; *p<0.05 versus the MTX group. (**G**) The percentage of damaged neurons. The data present the means ± standard error of the mean. (n=5 per group). #p <0.05 versus the control group; *p<0.05 versus the MTX group.

First, the open field test was run to test the locomotor ability and exploratory behavior of mice after different drug treatments, including MTX and CsA. As shown in Figure 1A, 1B, 1E, MTX treatment led to a significant decrease in the time traveled by mice in the center of the open field (F(4, 25)=3.87, *P* < 0.05), and this phenomenon was reversed by the intraperitoneal injection of CsA (*P* < 0.05). Neither MTX nor CsA caused a meaningful reduction in the total exploration distance covered by mice in the field over a period of 5 minutes, indicating that the locomotor activity of mice was not affected by the above treatments (F(4, 25)=0.41, *P* > 0.05).

As shown in Figure 1C, MTX notably decreased the freezing time in fear conditioning tests (*P* < 0.05), while CsA enhanced the post-MTX treatment freezing time (*P* < 0.05). However, applying KH-3, an inhibitor of HuR, reversed the neuroprotective effect of CsA by reducing the freezing time in the context fear test (F(4, 25)=11.05, *P* < 0.05).

To assess the preference for a novel object, the recognition index (RI) of the NOR test was calculated. Compared to the control group, mice had considerably lower recognition index scores in the presence of MTX (F(4, 25)=9.073, *P* < 0.05); CsA remarkably increased the RI scores of mice compared to MTX. However, KH-3 undid the impact of CsA (*P* < 0.05).

TUNEL staining of neurons in different regions of hippocampal tissues were used to verify the results of the behavior tests. As shown in Figure 1F, 1G, neuronal apoptosis in the CA1, CA3 and DG regions was drastically alleviated in MTX mice compared to the control group (F_CA1_(4, 21)=18.74; F_CA3_(4, 21)=32.65; F_DG_(4, 21)=21.24, *P* < 0.05). Similar to the results of the behavior tests, the percentages of TUNEL positive cells in CsA-treated mice decreased notably. However, CsA’s protective effects on neurons were reduced after KH-3 introduction (*P* < 0.05).

### Effects of CsA on the expression of HuR and ferritinophagy-related proteins in the hippocampus

**Figure 2:**
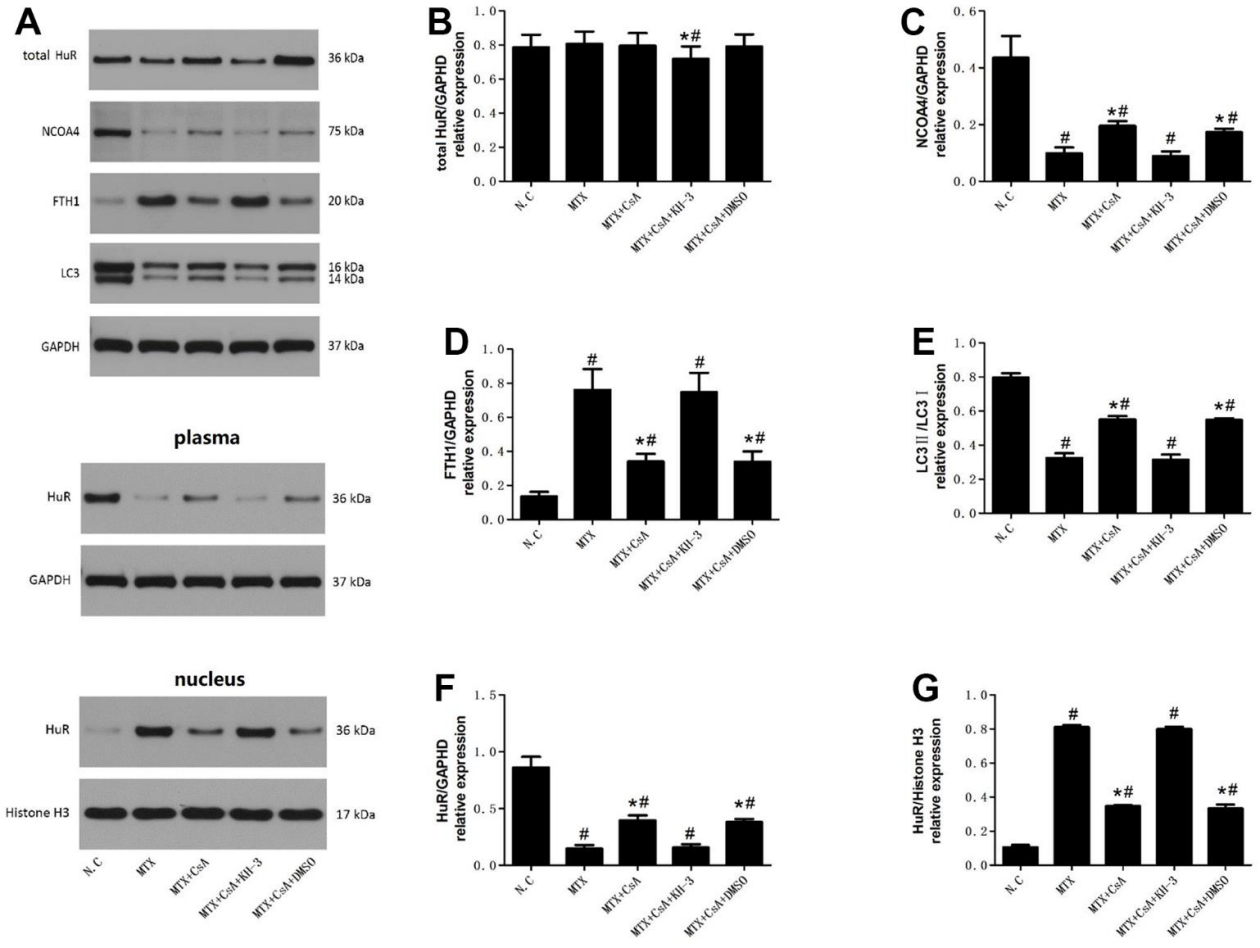
**Effects of CsA on the expression of HuR and ferritinophagy-related proteins in hippocampus.** (**A**) Representative blots of the total HuR, NCOA4, FTH1, LC3II/LC3I, HuR in the cytoplasm and of HuR in the nucleus in five groups of mice hippocampi. (**B**–**G**) Statistical results of the total HuR, NCOA4, FTH1, LC3II/LC3I, HuR in the cytoplasm and of HuR in the nucleus in five groups of mice hippocampi. The data present the means ± standard error of the mean. (n=3 per group). #p <0.05 versus the control group; *p<0.05 versus the MTX group.

Differences between HuR and ferritinophagy-related proteins in the hippocampal tissues of mice in each group were probed. The results are shown in Figure 2. In Figure 2A–2F, total HuR levels only decreased in the MTX+CsA+KH-3 group compared to the control group (Figure 2B, F(4, 10)=5.247, *P* < 0.05), with no significant changes registered in the other groups (*P* > 0.05). However, MTX treatment promoted the shuttling of HuR from the cytoplasm to the nucleus (Figure 2F, F(4, 10)=31.85; Figure 2G, F(4, 10)=43.22, *P* < 0.05), and intervention with CsA heightened the expression levels of cytoplasmic HuR compared to the MTX group (*P* < 0.05). As expected, there was also a decrease in the expression levels of nuclear HuR following CsA treatment. Meanwhile the expression levels of ferritinophagy-related proteins, such as NCOA4, and LC3II, declined too, and the differences were statistically considerable (Figure 2C, F(4, 10)=14.03; Figure 2E, F(4, 10)=65.75, *P* < 0.05). Relative to the control group, the expression levels of FTH1 increased extensively in the MTX group (Figure 2D, F(4, 10)=11.28, *P* < 0.05), while the levels of ferritinophagy-related protein, such as NCOA4, and LC3II were higher after CsA treatment than in the MTX group. In addition, the presence of CsA prompted a decrease in the levels of FTH1 (*P* < 0.05). However, KH-3 reversed these phenomena of CsA on the expression of ferritinophagy-related proteins in the hippocampus (*P* < 0.05).

### CsA prevents neuroinflammation in the hippocampus

**Figure 3:**
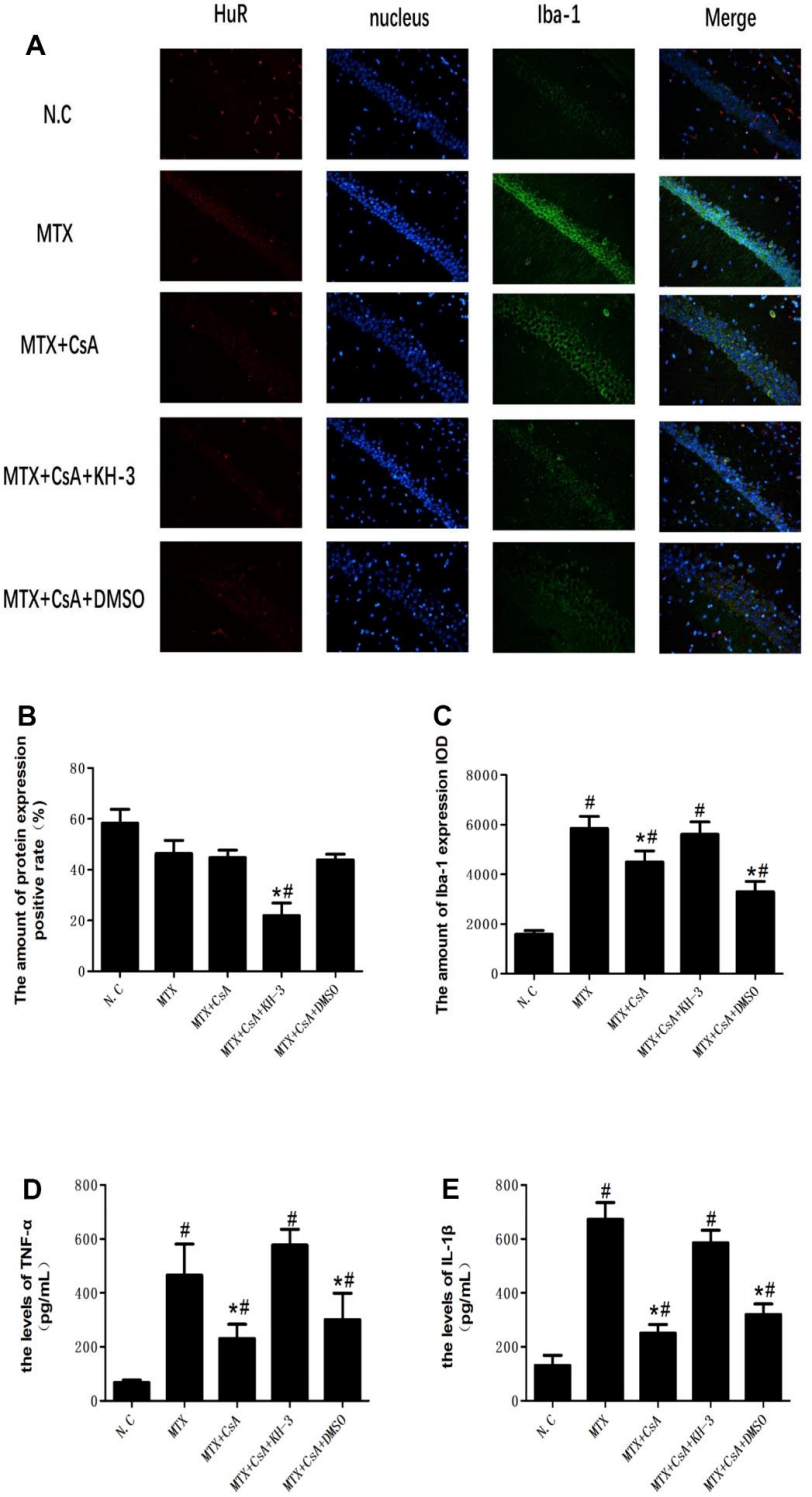
**Colocalization of HuR and Iba-1 in mice hippocampi with immunofluorescence.** (**A**) The colocalization of HuR and Iba-1 in mice hippocampi with immunofluorescence. (**B**) The amount of the HuR protein expression positive rate in the CA1 region of the hippocampus, as determined using immunofluorescence staining. (**C**) The intensity of Iba-1 in the CA1 region of the hippocampus, as determined using immunofluorescence staining. (**D**) The levels of TNF-α in five groups of mice hippocampi, as measured using ELISA. (**E**) The levels of IL-1β in five groups of mice hippocampi, as measured using ELISA. The data present the means ± standard error of the mean. (n=3 per group). #p <0.05 versus the control group; *p<0.05 versus the MTX group. Representative micrographs: ×400 magnification.

To measure the major pathological manifestation of neuroinflammation in the hippocampus, immunofluorescence was used to identify the colocalization of Iba-1 and HuR and assess related pro-inflammatory cytokines in mice hippocampi. As shown in Figure 3, MTX stimulated the Iba-1 immunoreactivity area in the hippocampus and increased the levels of TNF-α and IL-1β compared to the control group (Figure 3B, F(4, 10)=9.23; Figure 3C, F(4, 10)=17.87; Figure 3D, F(4, 10)=7.98; Figure 3E, F(4, 10)=9.16, *P* < 0.05). In contrast, CsA attenuated microglial activation by quietening the expression of Iba-1 and decreasing the levels of TNF-α and IL-1β in mice hippocampi (*P* < 0.05). However, KH-3 neutralized the effects of CsA on the expression of both Iba-1 and of HuR in mice hippocampi (*P* < 0.05).

### Ultrastructural mitochondrial changes in the hippocampus in different groups

**Figure 4:**
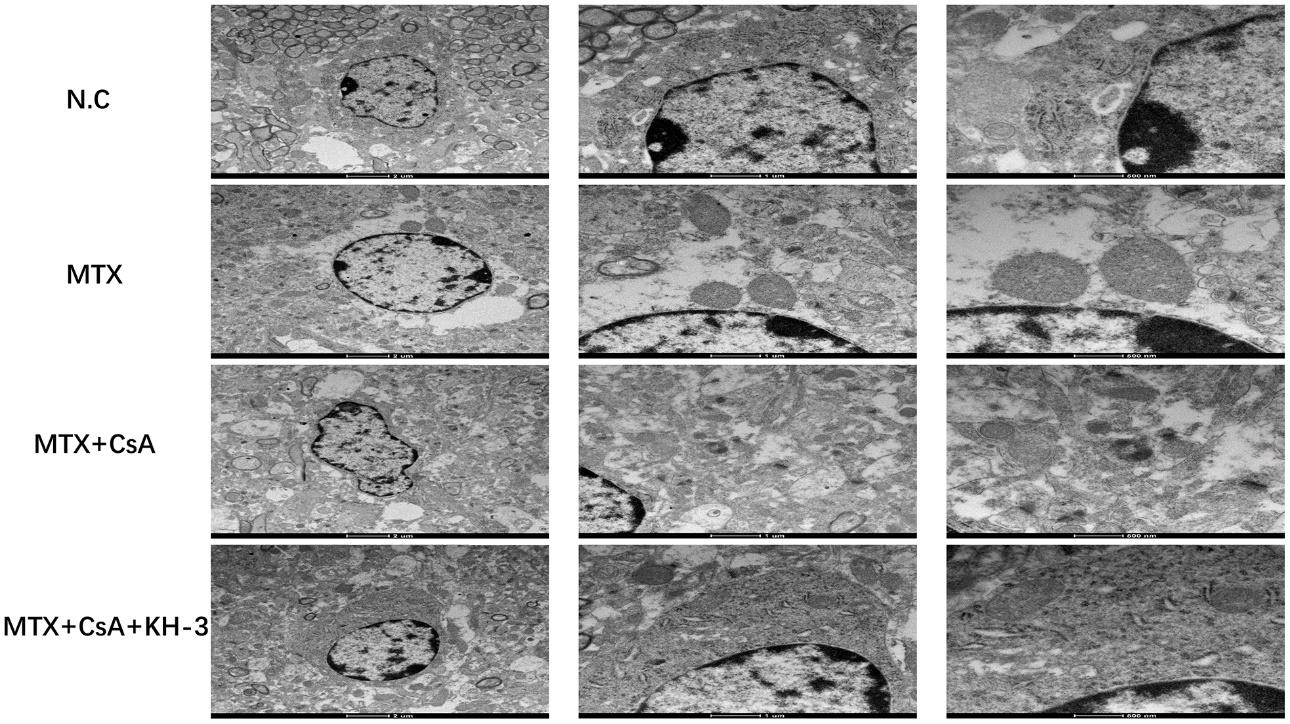
**Ultrastructural mitochondrial changes in the hippocampus in different groups.** Transmission electron microscopy images of hippocampal neuronal mitochondria from each group (scale bar = 2 um, 1 um and 500 nm from left to right, respectively).

As shown in Figure 4, which displays transmission electron microscopy results, MTX caused damages to the mitochondrial structure, such as mitochondrial swelling and dark mitochondrial matrix, in hippocampal tissues. Compared to the control group, the number of autophagosomes in the MTX group appeared to decrease, although statistical analysis was not possible. After CsA intervention, the morphology of mitochondria seemed to improve. However, the protective impact of CsA over mitochondria was halted by KH-3, including shrinkage of the mitochondrial membrane and a reduction in or the disappearance of mitochondrial cristae.

### CsA alleviated neuronal apoptosis in MTX-treated HT22 cells through HuR

**Figure 5:**
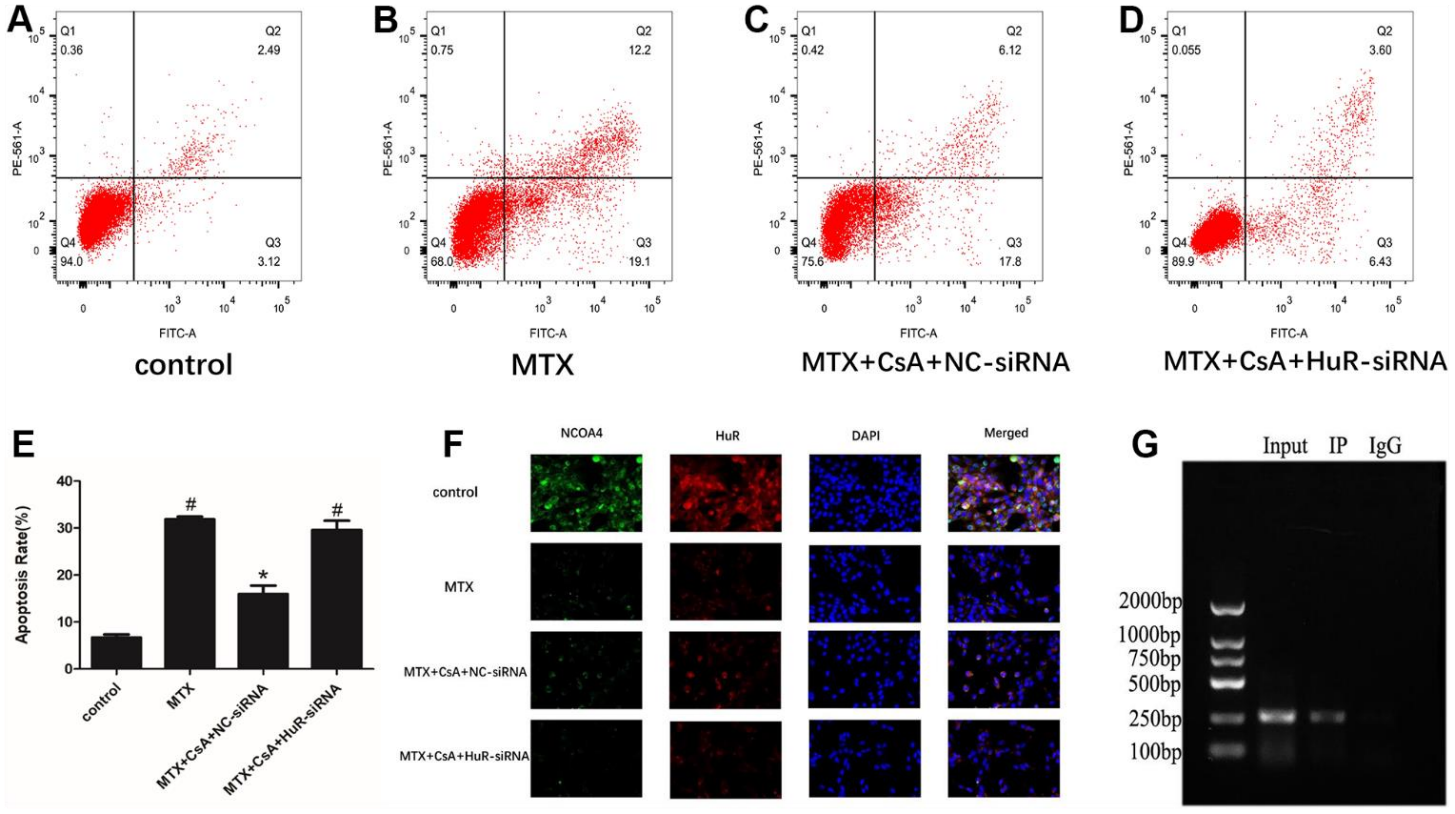
**CsA alleviated neuronal apoptosis in MTX-excited HT22 cells through HuR.** (**A**–**D**) Representative results on apoptotic cells in the Control, MTX, MTX+CsA+NC-siRNA, and MTX+CsA+HuR-siRNA groups, as evaluated using flow cytometry. (**E**) Flow cytometry analysis of HT22 cells in the Control, MTX, MTX+CsA+NC-siRNA, and MTX+CsA+HuR-siRNA groups. (n=3 per group). #p <0.05 versus the control group; *p<0.05 versus the MTX group. (**F**) HT22 cell, as captured after co-staining with HuR and NCOA4. In the representative images, HuR and NCOA4 are shown in red and green, respectively. Representative micrographs: ×400 magnification. (**G**) HR22 cell lysis immunoprecipitation with anti-HuR antibody or control IgG.

In order to test the effect of HuR in MTX-treated HT22 cells, siRNA was used to knock down HuR in these cells. HuR siRNA and its negative control (NC) were transfected into the test and control HT22 cells, respectively. As shown in Figure 5A–5E, compared to the control group, MTX treatment significantly increased neuronal apoptosis (F(3, 8)=69.93, *P* < 0.05); relative to the MTX group, CsA reduced neuronal apoptosis in the MTX-treated HT22 cells (*P* < 0.05), exhibiting its neuroprotective effects. However, the addition of HuR-siRNA put a stop to CsA’s protective effect (*P* < 0.05).

Immunofluorescence co-localization revealed that the expression of ferritinophagy receptor-NCOA4 and HuR in HT22 cells was higher after CsA treatment than after exposure to MTX (*P* < 0.05, Figure 5F). However, cell transfection with HuR-siRNA saw a marked decrease in the expression of HuR and the abolition of the CsA-induced NCOA4 expression in HT22 cells (*P* < 0.05, Figure 5F), implying that the inhibition of HuR eliminated CsA’s protection of MTX-excited HT22 cells through NCOA4-mediated ferritinophagy. Furthermore, the assessment of the ability of HuR to bind to NCOA4 mRNA with an anti-HuR antibody or IgG using the RNA immunoprecipitation (IP) assay showed HuR binding to NCOA4 mRNA (Figure 5G).

### The CsA-mediated activation of HuR lessens MTX-induced cytotoxicity and inflammatory injuries in HT22 cells via NCOA4-mediated ferritinophagy

**Figure 6:**
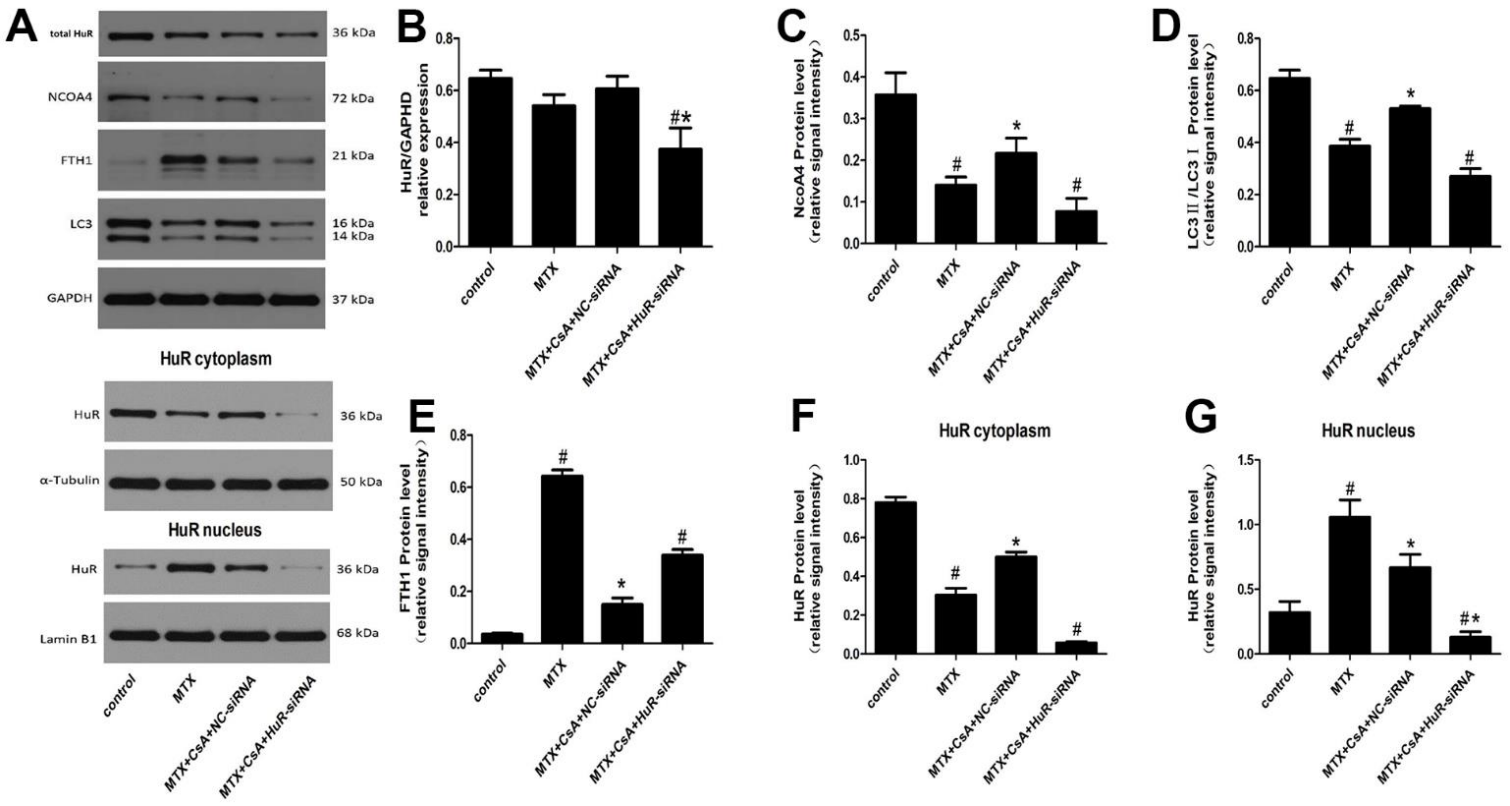
(**A**) Representative blots of the total HuR, NCOA4, FTH1, LC3II/LC3I, HuR in the cytoplasm and of HuR in the nucleus in HT22 cells after different treatments. (**B**–**G**) Statistical results of the total HuR, NCOA4, FTH1, LC3II/LC3I, HuR in the cytoplasm and of HuR in the nucleus in HT22 cells with different treatments. The data present the means ± standard error of the mean. (n=3 per group). #p <0.05 versus the control group; *p<0.05 versus the MTX group.

Per Figure 6A, 6B, 6F, 6G, total HuR levels only decreased in the MTX+CsA+HuR-siRNA group (Figure 6B F(3, 8)=4.83, *P* < 0.05) compared to the control group, with no significant changes noted in the other groups. Also, MTX treatment caused HuR translocation from the cytoplasm to the nucleus (*P* < 0.05). In contrast, the CsA group engineered a large increase in cytoplasmic HuR expression compared to the MTX group (Figure 6F, F(3, 8)=136.1; Figure 6G, F(3, 8)=17.68, *P* < 0.05), suggesting that CsA treatment can promote the translocation of HuR from HT22 cell nuclei to their cytoplasm.

Western blot analyses also revealed that CsA caused an elevation in the expression of ferritinophagy receptor-NCOA4 and LC3II in HT22 cells compared to cells treated with MTX (Figure 6C, F(3, 8)=10.32; Figure 6D, F(3, 8)=40.32; Figure 6E, F(3, 8)=173.5, *P* < 0.05, Figure 6). By contrast, ferritinophagy substrate-FTH1 was substantially reduced after treatment with CsA (*P*<0.05). However, the transfection of cells with HuR siRNA resulted in the remarkable down-regulation of the expression of LC3II and blunted the CsA-induced NCOA4 expression in HT22 cells (*P*<0.05, Figure 6A–6E). At the same time, the levels of FTH1 were up-regulated by HuR siRNA treatment, revealing that the inhibition of HuR eliminated CsA’s protection of MTX-stimulated HT22 cells, implying that NCOA4-mediated ferritinophagy was involved in the neuroprotection of HuR in the brain.

Furthermore, the assessment of the effects of CsA on neuroinflammation was carried out via measurement of the concentrations of TNF-α and IL-1β in the hippocampus. As illustrated in Supplement 1, the levels of TNF-α and IL-1β increased considerably after MTX stimulation (F_TNF-α_(3, 8)=18.36; F_IL-1β_(3, 8)=7.99, *P*<0.05). Coincubation with CsA tended to markedly alleviate the concentrations of TNF-α and IL-1β compared to incubation with MTX alone (*P*<0.05). However, HuR siRNA silenced CsA’s reversal of MTX-induced inflammation in HT22 cells, indicating that the CsA-mediated activation of HuR could attenuate MTX-induced inflammation in HT22 cells.

## DISCUSSION

With increasingly more cancer patients receiving chemotherapy, the side effects of the treatment are becoming more apparent, especially in terms of cognitive impairment. Therefore, exploring the mechanism of CICI and finding effective ways to prevent and treat CICI are of great significance. This study found MTX capable of causing neuronal apopotosis and neuroinflammation in the hippocampus of mice, leading to impaired cognitive behavior. Treatment with CsA drastically alleviated this MTX-induced cognitive function impairment, while the impact of the HuR inhibitor, KH-3, on CsA’s activity showed that HuR participated in the neuroprotective action of CsA, which is possibly involved in NCOA4-mediated ferritinophagy.

Evidence shows that oxidative damage, mitochondrial dysfunction, and loss of choline-containing biomolecules constitute the triangle of death of neurons in mice with CICI [12–14]. Targeting mitochondrial dysfunction, many researchers have demonstrated that mitophagy is involved in the pathophysiological processes of CICI. In this investigation, MTX was also shown as being able to cause abnormal mitochondrial morphology and decreased mitophagy in mice hippocampi. Along with the improvement of mitochondrial morphology and function, CsA alleviated MTX-induced CICI by halting neuronal apoptosis. Consistent with this finding, a previous study found that Pifithrin-μ offered a tractable therapeutic strategy to limit this common side-effect of chemotherapy by inhibiting mitochondrial p53 accumulation and preventing neuronal mitochondrial function. Additionally, the current research revealed that CICI was characterized by elevated levels of pro-inflammatory cytokines in the hippocampus, which matches findings from many other CICI analyses.

On the basis of autophagy, ferritinophagy, as a new type of autophagy, occurs in more and more pathophysiological processes of many diseases, such as diabetes [15], spinal cord injury [16] and neurodegenerative diseases [17]. Using quantitative proteomics, some probes have established NCOA4 as a cargo receptor for selective autophagy and modulated ferritin degradation in autophagosomes [18]. This process is specifically involved with ferritin to release intracellular free iron. Recently, evidence has surfaced of the identification of the vital role of NCOA4-mediated ferritinophagy in the pathogenesis of central nervous system diseases. For instance, Li et al. [19] showed that NCOA4-mediated ferritinophagy plays a vital role in early cerebral ischemia-reperfusion injury (CIRI) in mice and can be an effectively targeted to prevent and treat CIRI. To explore the relationship between NCOA4 and the cGAS-STING pathway, inquiries have additionally administered a cGAS inhibitor to mice with CIR and overexpressed NCOA4, revealing that the activation of the cGAS-STING pathway exacerbates CIRI, potentially through the modulation of NCOA4-mediated ferritinophagy. NCOA4-mediated ferritinophagy has also been identified in chronic unpredictable mild stress mice and corticosterone-treated HEK-293T cell lines [16]. Notably, the present assessment uncovered NCOA4-mediated ferritinophagy in MTX-induced cognitive impairment in mice and MTX-excited HT22 neuronal cells. However, the results of this current study differ in some way from those of existing investigations. Unlike other analyses, in which NCOA4 was highly expressed in the model group, NCOA4 expression in the MTX group in this study was low. Also, the present evaluation found the expression of NCOA4 to correlate positively with the autophagy-related protein LC3II and negatively with FTH1. MTX treatments inhibited the expression of NCOA4 and LC3II, leading to the accumulation of FTH1. These findings are similar to some in the past. Per Qin et al. [5], NCOA4 mediates the degradation of ferritin in ZnONPs-stimulated endothelial cells, with exposure to ZnONPs inducing a decrease in FTH1 and an increasing tendency followed by the decreased expression of NCOA4 in vascular endothelial cells. Additionally, NCOA4 silencing disrupts ferritin turnover and is cytotoxic when cells are iron deficient [20]. Moreover, the authors of the present inquest found previously that MTX induces neurotoxicity and inflammation in HT22 cells through intervening in NCOA4-mediated ferritinophagy and the accumulation of FTH1, presenting a potential therapeutic strategy for MTX-induced CICI [21].

CsA, as an immunosuppressive agent, exhibits neurotrophic and neuroprotective properties in animal models of CNS disorders, including stroke [22], traumatic brain injury [8], and Parkinson’s disease [5, 23]. Its neuroprotective mechanism includes preserving mitochondrial integrity, calcineurin inhibition and the activation of endogenous neural precursor cells. For example, Tajiri et al. [24] demonstrated that CsA maintains mitochondrial integrity by upregulating DJ-1, which is a beneficial therapeutic strategy for a stroke in mice. Reportedly, CsA treatment activates the neural precursor cell population, promotes the migration of neural precursor cells to the site of injury, and leads to the recovery of cognitive function after long-term treatment. In this study, CsA also relieved MTX-induced cognitive function impairment by inhibiting neuroinflammation in the hippocampus, a finding consistent with results of other inquisitions. however, CsA’s specific underlying mechanisms must still be identified.

According to recent findings, CsA engineers the cytoplasmic translocation of HuR and promotes the phosphorylation of HuR. HuR, an RNA-binding protein, regulates gene expression by binding to target genes’ 3' UTR region and is highly expressed in the liver, heart and central nervous system. Research has shown that hepatic HuR modulates lipid homeostasis in response to high-fat diets [25]. HuR is a key gatekeeper of liver homeostasis and prevents hepatocellular carcinoma and non-alcoholic fatty liver disease-related fibrosis, suggesting that the HuR-dependent network could be used therapeutically. However, whether HuR is a friend or foe in the field of human cancer remains a matter of debate, even if previous evidence shows that targeting the inhibition of HuR presents beneficial effects against glioma [26], prostate cancer [27], breast cancer [28] and pancreatic cancer [29]. Regarding the central nervous system, HuR is highly expressed in the neocortex during developmental stages, and its absence destabilizes the laminar structure of the neocortex, indicating that HuR mRNA metabolism promotes the cell motility of migrating mouse neurons [30]. In addition, the loss of HuR allegedly gives rise to defective ependymal cells and hydrocephalus, pointing to the essential role of HuR in the posttranscriptional regulation of ependymal cell development [31]. In the present study, CsA did not alter the expression levels of HuR; however, it promoted the shuttling of HuR from the nucleus to the cytoplasm, providing a possible new mechanism for its neuroprotective effects. The present inquiry also revealed the relationship between HuR and NCOA4-mediated ferritinophagy in CICI. CsA mitigated CICI and enhanced the expression of NCOA4 by improving the shuttling of HuR from the nucleus to the cytoplasm, accompanied by reduced neuronal apoptosis, and neuroinflammation. Inhibiting the expression of HuR with KH-3 blunted the protective effects of CsA, pointing to the complex and multiple roles of HuR in human diseases.

## CONCLUSIONS

In summary, this investigation confirmed the neuroprotective role of CsA in CICI, with CsA’s yet undetermined underlying mechanisms possibly involved in the translocation of HuR. Intervening in the translocation of HuR during CICI could mitigate neruoinflammation and neuronal apoptosis via NCOA4-mediated ferritinophagy and, thus, alleviate cognitive impairment in mice with CICI.
